# Author Correction: Ultra-thin high-efficiency mid-infraredtransmissive Huygens meta-optics

**DOI:** 10.1038/s41467-018-04890-6

**Published:** 2018-06-14

**Authors:** Li Zhang, Jun Ding, Hanyu Zheng, Sensong An, Hongtao Lin, Bowen Zheng, Qingyang Du, Gufan Yin, Jerome Michon, Yifei Zhang, Zhuoran Fang, Mikhail Y. Shalaginov, Longjiang Deng, Tian Gu, Hualiang Zhang, Juejun Hu

**Affiliations:** 1 State Key Laboratory of Electronic Thin Films and Integrated Devices, National Engineering Research Center of Electromagnetic Radiation Control Materials, University of Electronic Science and Technology of China, Chengdu, Sichuan 611731 China; 2 Department of Materials Science & Engineering, Massachusetts Institute of Technology, Cambridge, MA 02139 USA; 3 School of Information and Science Technology, East China Normal University, Shanghai, 200062 China; 4 Department of Electrical & Computer Engineering, University of Massachusetts Lowell, Lowell, MA 01854 USA

Correction to: *Nature Communications* 10.1038/s41467-018-03831-7, published online 16 April 2018

The original version of this Article omitted the following from the Acknowledgements:

“J.D. and H. Zhang acknowledge initial funding for design of the meta-atoms provided by the National Science Foundation under award CMMI-1266251. Z.L. and H. Zheng contributed to the Device Fabrication section and were independently funded as visiting scholars by the National Natural Science Foundation of China under award 51772042 and the “111” project (no. B13042) led by Professor Huaiwu Zhang. Later work contained within the Device Modeling and Device Characterization sections and some revisions to the manuscript were funded under Defense Advanced Research Projects Agency Defense Sciences Office (DSO) Program: EXTREME Optics and Imaging (EXTREME) under Agreement No. HR00111720029. The authors also acknowledge fabrication facility support by the Harvard University Center for Nanoscale Systems funded by the National Science Foundation under award 0335765. The views, opinions and/or findings expressed are those of the authors and should not be interpreted as representing the official views or policies of the Department of Defense or the U.S. Government.”

This has been corrected in both the PDF and HTML versions of the Article.

