# Supplementary material for: Ultra-thin high-efficiency mid-infrared transmissive Huygens meta-optics
Source: Nat Commun. 2018 Apr 16;9:1481. doi: 10.1038/s41467-018-03831-7 (PMC5902483; doi:10.1038/s41467-018-03831-7)
Supplement: Supplementary file 1 — Supplementary Information [file 41467_2018_3831_MOESM1_ESM.pdf]

Supplementary Information

Ultra-thin, High-efficiency Mid-Infrared Transmissive Huygens Meta-Optics

Zhang, Ding, Zheng, et al

Supplementary Note 1 – Huygens metasurface (HMS) in a nutshell

Supplementary figure 1 illustrates a schematic of a dielectric Huygens metasurface, where an *x*-polarized plane wave is normally incident on the metasurface and transmitted through with arbitrary wavefront. Independent field distributions stipulated in the two regions of space (regions 1 and 2 in Supplementary Fig. 1) are separated by the metasurface. Since the fields are generally discontinuous at the surface *S* (at *z* = 0), fictitious electric and magnetic surface currents are needed on the surface to fulfill the boundary conditions according to the equivalent principle[1]:

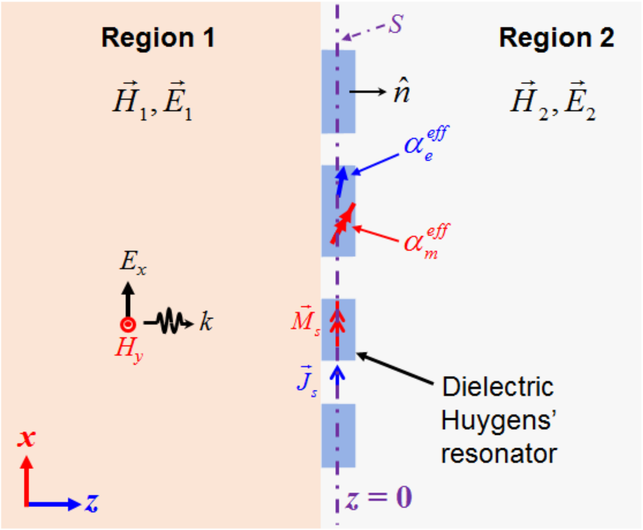

Supplementary Figure 1. Schematic of a dielectric Huygens metasurface. The metasurface satisfies the boundary condition between regions 1 and 2 by constructing equivalent electric and magnetic surface currents. The high-index dielectric Huygens' resonators can support both electric and magnetic resonances.

$$\vec{J}_s = \hat{n} \times (\vec{H}_2 - \vec{H}_1) \qquad (1)$$

$$\vec{M}_s = -\hat{n} \times (\vec{E}_2 - \vec{E}_1) \qquad (2)$$

The surface currents can be related to the surface electric and magnetic polarizabilities ($\alpha_e^{eff}, \alpha_m^{eff}$) by averaging the fields of the electric/magnetic dipole moments on S [2]:

$$\vec{J}_s = j\omega\alpha_e^{eff} \bullet \vec{E}_{t,av}\Big|_S \qquad (3)$$

$$\vec{M}_s = j\omega\alpha_m^{eff} \bullet \vec{H}_{t,av}\Big|_S \qquad (4)$$

where a time dependence $e^{j\omega t}$ is assumed, *E*/*H* represent the average electric/magnetic fields tangential to the surface *S*, and ω denotes the angular frequency. Furthermore, the complex reflection (*R*) and transmission (*T*) coefficients of a normally incident plane wave can be related to the polarizabilities of a periodic metasurface via[3]:

$$j\omega\alpha_e^{eff} = \frac{2(1-T-R)}{\sqrt{\mu/\varepsilon}(1+T+R)} \qquad (5)$$

$$j\omega\alpha_m^{eff} = \frac{2\sqrt{\mu/\varepsilon}(1-T+R)}{(1+T-R)} \qquad (6)$$

where μ and ε are the permeability and permittivity of the free space, respectively. According to Eqs. 5 and 6, it can be concluded that arbitrary phase modulation and near-unity transmittance can be achieved by engineering the dielectric resonators with suitable polarizabilities[4,5].

Supplementary Note 2 – Transmission properties of rectangular meta-atoms

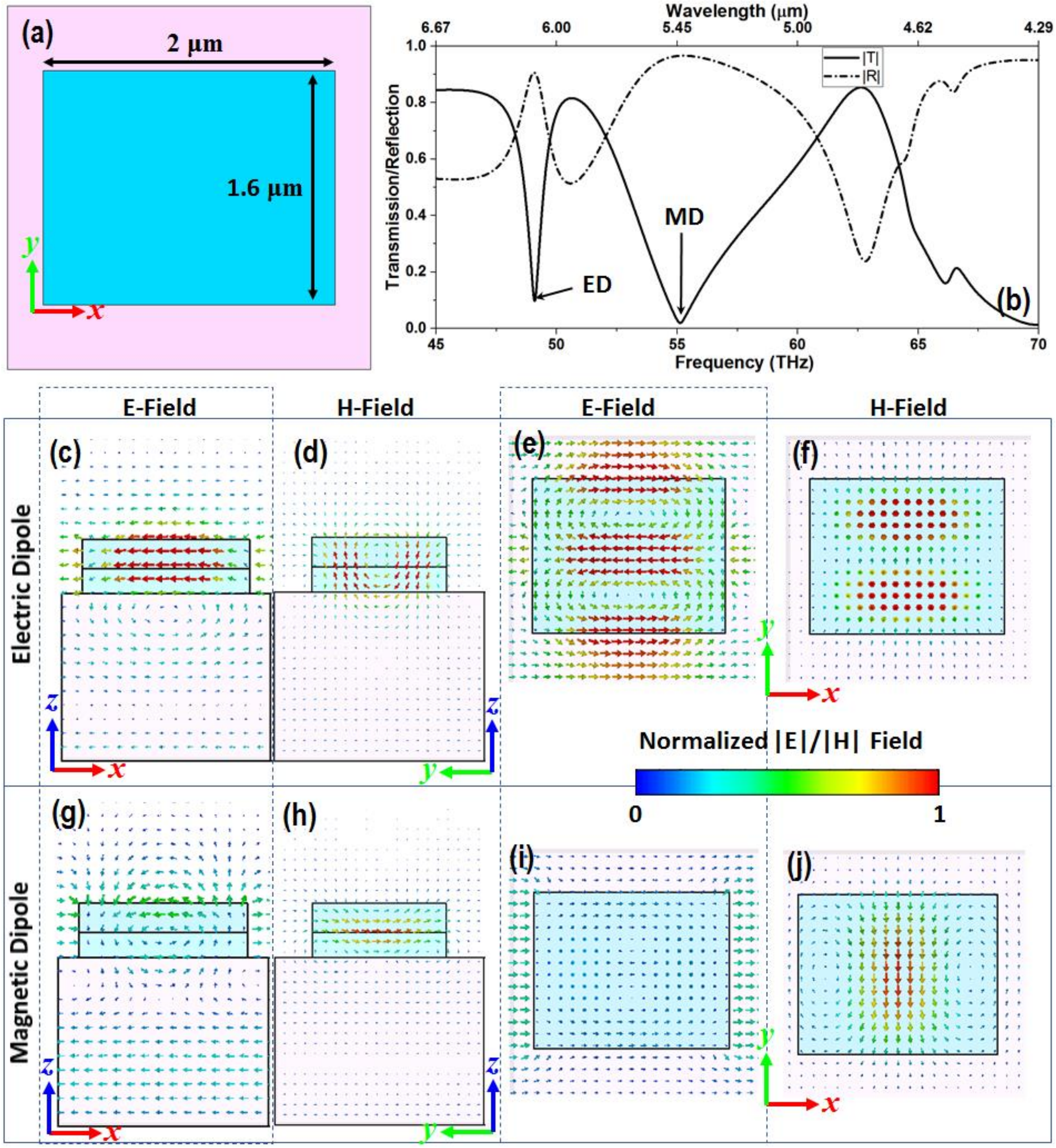

Supplementary Figure 2. (a) Schematic top-view of a rectangular PbTe-on-CaF_2 resonator; (b) transmission and reflection spectra of an infinite array formed by a periodic arrangement of the unit cell in (a), showing two spectrally separated resonances (ED and MD); (c-f) field distributions for the ED resonance at different cutting planes; (g-j) field distributions for the MD resonance at different cutting planes.

As discussed in Supplementary Note 1, a salient feature of a HMS is collocated electric dipole (ED) and magnetic dipole (MD) resonances within its meta-atoms. In this section, we analyze the electric and magnetic resonant behavior of the rectangular PbTe-on-CaF_2 meta-atoms (Fig. 1a),

and describe methods to engineer optical transmission properties of the meta-atoms via tuning the ED and MD resonances. We note that the high refractive index of PbTe (n ~ 5) in the mid-IR regime facilitates meta-atom design optimization to conceive matched electric and magnetic dipole resonances.

Supplementary figure 2 illustrates the transmission properties of an infinite array of rectangular PbTe resonators situated on top of a semi-infinite CaF_2 substrate. The simulation was performed using the CST Microwave Studio, assuming an *x*-polarized incident plane wave propagating along the *z*-axis. The transmission spectrum plotted in supplementary figure 2b clearly shows two well-separated dips corresponding to the resonances of ED and MD, which are the two lowest multipolar resonances of interest. Supplementary figures 2c-f and Figs. 2g-j plot the field distributions at different cutting planes for the ED and MD resonance, respectively. Supplementary figures 2c-d validate the electric resonance behavior with the E-field (in the *x*-*z* plane) concentrated at the center of the resonator and vortex-like H-field (in the *y*-*z* plane) surrounding the E-field. Supplementary figures 2e-f show the top-view (in the *x*-*y* plane) of the E-field and H-field at the interface between top and bottom layers of PbTe at $z = 0.3$ µm, respectively. Supplementary figure 2e indicates that the E-field vector goes from right end to the left end in the middle and loops back from both top and bottom ends. On the other hand, Supplementary Figs. 2g-j demonstrate the magnetic resonance behavior with the H-field (in the *y*-*z* plane) concentrated at the center of the resonator and vortex-like E-field (in the *x*-*z* plane) surrounding the H-field. The displacement current loop resulting from the vortex E-field generates the MD resonance.

In the example shown in Supplementary figure 2, the electric and magnetic resonances are well-separated spectrally and can be easily identified. The two resonances shift at different paces when the resonator size varies, as shown in Supplementary Figs. 3a-c. Supplementary figure 3a shows that the ED resonance is red-shifted towards longer wavelength when L_y increases while fixing $L_x = 2$ µm. Meanwhile, the spectral position of the magnetic resonance remains almost unchanged. In contrast, Supplementary figure 3b illustrates that the magnetic resonance is red-shifted when increasing L_x and fixing $L_y = 1.2$ µm, whereas little spectral shift is observed for the electric resonance despite its transmission amplitude change. The ability to independently tune the ED and MD resonances permits us to engineer the meta-atom's transmission properties (phase and amplitude) by varying the dimensions of the rectangular block.

In Supplementary Figs. 3a and 3b, the ED and MD resonances are spectrally separated, and the transmittances at the target wavelength ($\lambda_0 = 5.2$ µm) are low. In comparison, Supplementary figure 3c plots the transmission spectra of resonators with partially overlapping ED and MD resonances, which leads to improved transmission at the wavelength of interest. Ideally, when the two resonances completely overlap, unity transmittance can be achieved[6,7]. In our HMS, the meta-atoms are designed to be slightly off-resonance to obtain desired phase shifts (between 0 to 2π) while maintaining high transmission efficiencies.

Figures 1b and 1c plot numerically simulated rectangular resonator transmission phase and amplitude as functions of L_x and L_y at $\lambda_0 = 5.2$ µm. The choice of the dimension range (0.5 – 2 µm) limits the aspect ratio of the meta-atom structures to be less than 1.3, which significantly simplifies the fabrication process and improves processing yield. The same data set in Figs. 1b and 1c are also plotted in Supplementary figure 3d, which shows the phase distribution with corresponding transmission amplitude for the rectangular cells. Optimal meta-atom designs presented in Fig. 1d correspond to the data points which give rise to the maximum transmittance in Supplementary figure 3d. As we allude to in the main text, Supplementary figure 3d and Fig. 1d illustrate the presence of a "low-efficiency gap" where the rectangular meta-atoms suffer from low optical

transmittance. Such low-efficiency gaps also account for the limited efficiencies of previously reported dielectric HMS devices[8-11], which comprise meta-atoms of a single type of geometry with only size variation.

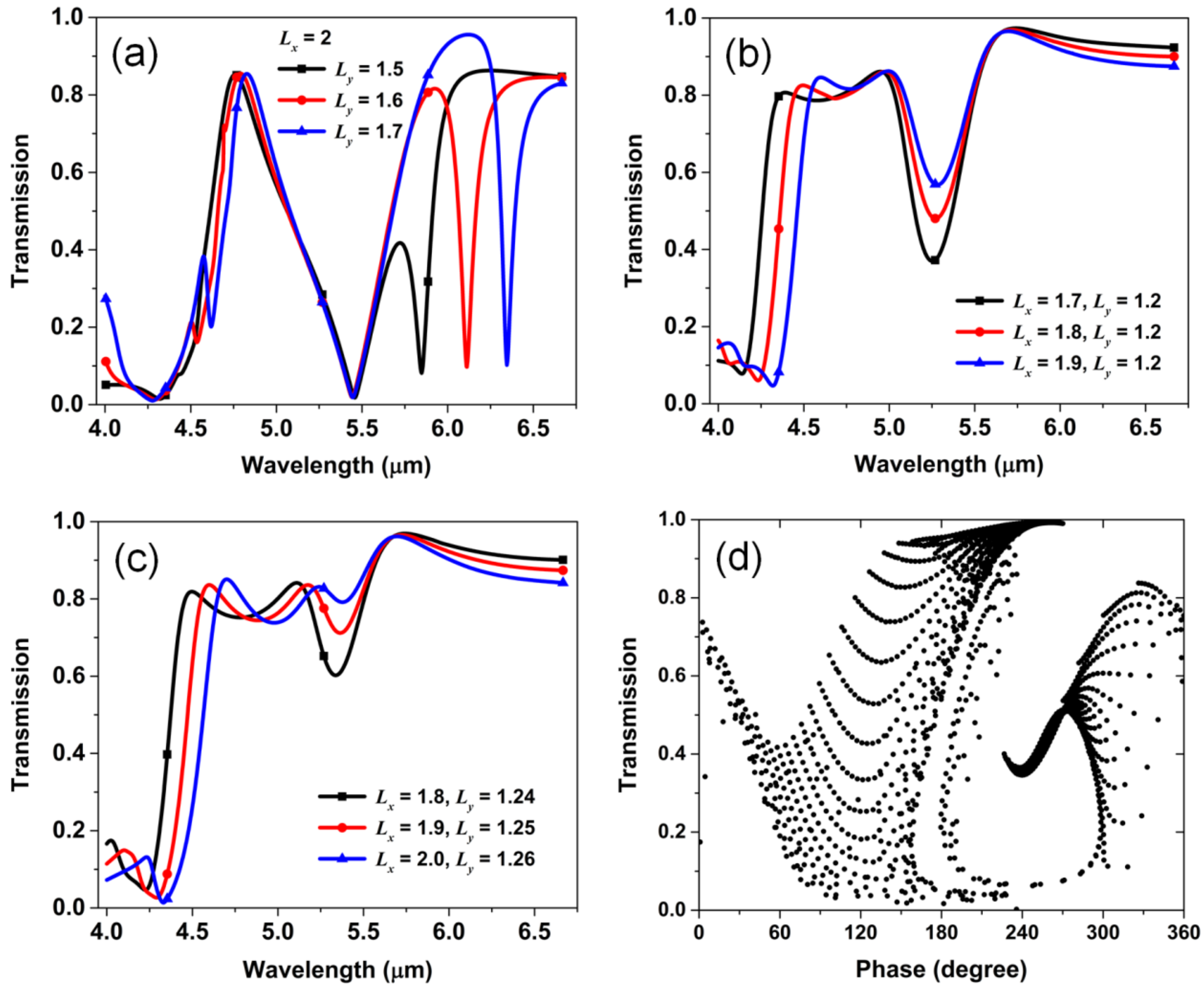

Supplementary Figure 3. Evolution of transmission spectra of rectangular meta-atoms showing two spectrally separated resonances: (a) when varying L_y while keeping L_x constant; and (b) when changing L_x and fixing L_y; (c) Transmission spectra of rectangular resonators with two partially overlapping resonances; (d) scatter diagram of the rectangular meta-atom transmission phase and amplitude shown in Figs. 1b and 1c: upper envelope of the diagram corresponds to the optimized meta-atom performance shown in Fig. 1d. The length unit is micron in the figures.

Supplementary Note 3 – Transmission properties of H-shaped meta-atoms

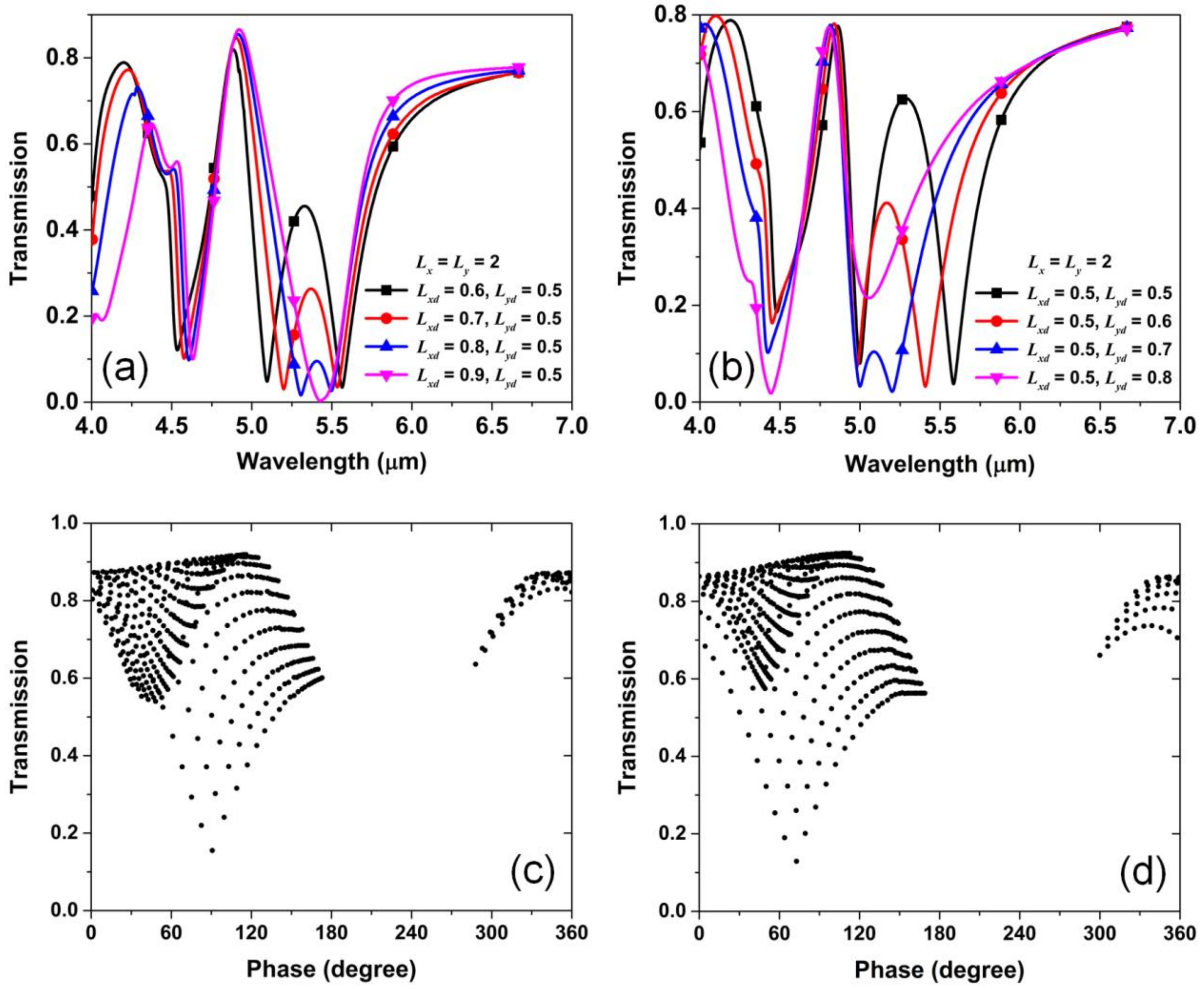

Supplementary Figure 4. Evolution of transmission spectra of H-shaped meta-atoms showing two spectrally separated resonances: (a) when varying L_{yd} while keeping L_{xd} constant; and (b) when changing L_{xd} and fixing L_{yd}; (c, d) scatter diagrams of the H-shaped meta-atom transmission phase and amplitude. In (c), L_x = 2 μm, L_y = 1.7 μm, L_{xd} is varied from 0.5 to 1 μm, and L_{yd} from 0.5 to 1.2 μm, both with a step size of 0.025 μm. In (d), L_x = 1.8 μm, L_y = 2 μm, while L_{xd} and L_{yd} are both varied from 0.5 to 1.1 μm with a step size of 0.025 μm. The length unit is micron in the figures.

To overcome the "low-efficiency gap" issue discussed in Supplementary Note 2, we devised a new type of unit cell, i.e., the H-shaped resonator. Here we first illustrate that both electric and magnetic resonances can be spectrally tailored by varying L_{xd} and L_{yd}, the width and length of the dielectric bar as shown in Fig. 1e. Supplementary figure 4a indicates that the magnetic resonance is considerably more sensitive to the change of L_{xd} (with fixed L_{yd}) compared to the electric resonance. On the other hand, Supplementary figure 4b reveals that the electric resonance blue shifts with increasing L_{yd} (with fixed L_{xd}) while the magnetic resonance barely shifts. Supplementary figures 4c and 4d plot the simulated transmission amplitude and phase of H-shaped resonators with varying dimensions. In both cases, the H-shaped resonators exhibit high transmission (> 0.85) within the phase angle range of 0 to 130 degrees, which fully bridges the "low-efficiency gap" of

the rectangular resonators. Thus, a full 2π phase coverage with high transmission can be realized with our two-component HMS design which include both rectangular and H-shaped resonators to generate arbitrary wavefront. Supplementary table 1 summarizes the dimensions of the meta-atoms used in our HMS meta-optical devices. Given that the PbTe meta-atoms exhibit slanted sidewalls, the dimensions listed in Table S1 are measured at the bottom of the PbTe blocks.

Supplementary Table 1. Dimensions of meta-atoms used in the meta-optical devices

Meta-atom number	1	2	3	4	5	6	7	8
L_x (L_{xd})	2.0 (0.6)	1.8 (0.6)	2.0 (0.7)	2	1.78	1.38	0.62	2
L_y (L_{yd})	1.7 (0.9)	1.9 (0.8)	1.6 (0.7)	0.78	0.7	0.66	0.52	1.26

To gain further insight into operation of the H-shaped resonators, we investigated the properties of their electric and magnetic dipole resonances. Supplementary figure 5a shows the top-view of an exemplary H-shaped PbTe resonator. Its transmission spectrum displayed in Supplementary figure 5b clearly reveals two resonance dips corresponding to ED and MD resonances. Supplementary figures 5c-f and Figs. 5g-j illustrate the field profiles at different cutting planes at ED and MD resonances, respectively. Supplementary figures 5c and 5d suggest that at ED resonance, E-field in the *x*-*z* plane is concentrated at the center of the resonator with vortex-like H-field in the *y*-*z* plane, similar to the case of rectangular resonators (Supplementary Figs. 2c and 2d). Supplementary figures 5h and 5g demonstrate that the magnetic resonance features H-field in the *y*-*z* plane confined at the center of the resonator whereas vortex-like E-field in the *x*-*z* plane surrounds the H-field, which again closely resembles the case of rectangular resonators (Supplementary Figs. 2h and 2g). Supplementary figures 2e and 2f show the *x*-*y* section view of the ED resonance E-field and H-field at the interface between the top and bottom PbTe layers at z = 0.3 µm, respectively. Compared to Supplementary figure 2e, Supplementary figure 5e shows a markedly different E-field distribution with four vortex-like loops emerging in the corners. Supplementary figures 2i and 2j portray the *x*-*y* section view of the MD resonance E-field and H-field, respectively. Compared to Supplementary figure 2j, Supplementary figure 5j exhibits a similar H-field distribution although the H-field becomes a lot stronger at the right and left ends. Analogous to the rectangular resonators, the H-shaped resonators also support ED and MD resonances essential for HMS operation, but the distinctive field distributions account for the observed different transmission properties.

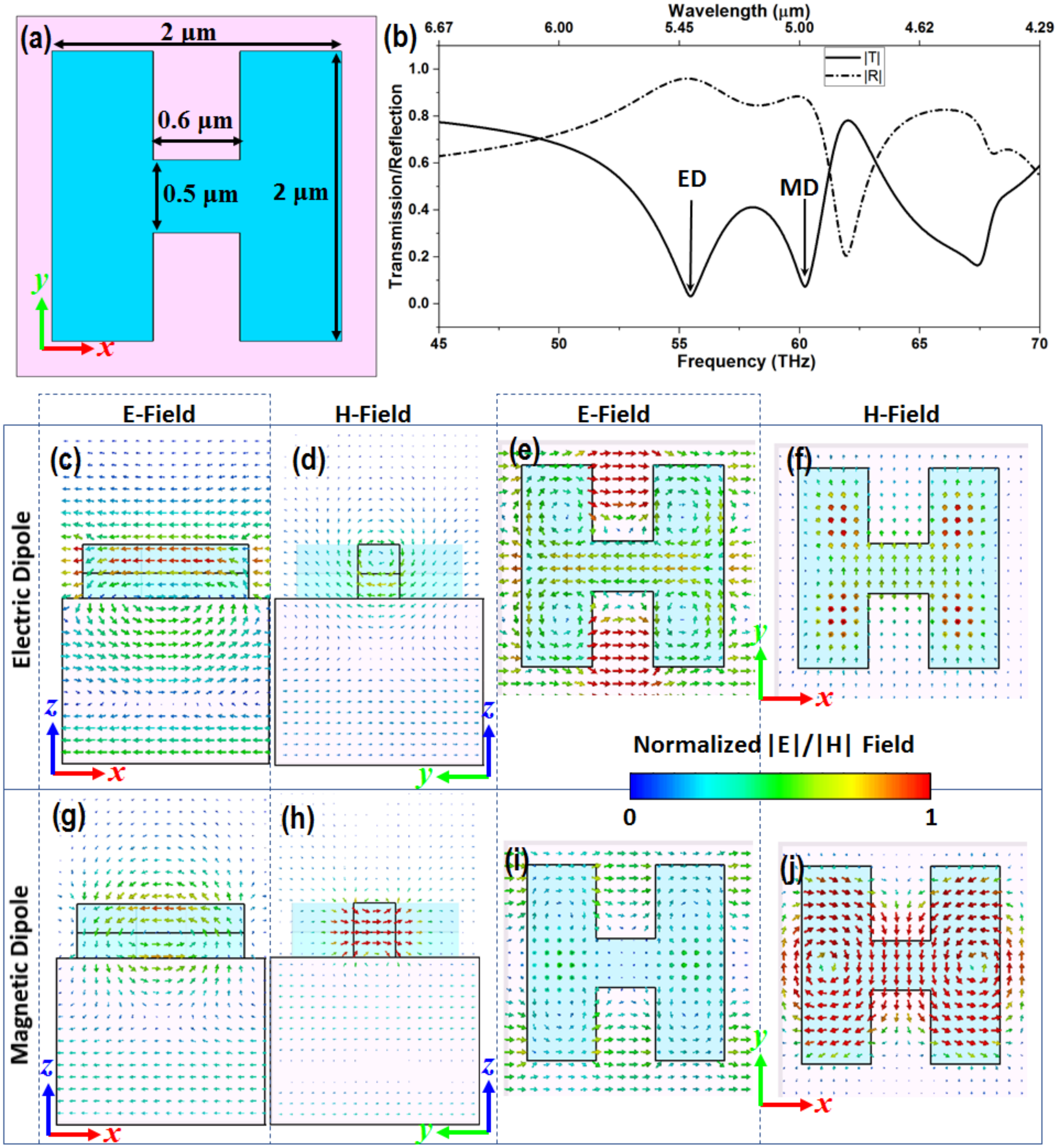

Supplementary Figure 5. **(a) Schematic top-view of a H-shaped PbTe-on-CaF_2 resonator; (b) transmission and reflection spectra of an infinite array formed by a periodic arrangement of the unit cell in (a), showing two spectrally separated resonances (ED and MD); (c-f) field distributions for the ED resonance at different cutting planes; (g-j) field distributions for the MD resonance at different cutting planes.**

Supplementary Note 4 – Beam deflector measurement protocols

Supplementary figure 6 illustrates the testing setup for the metasurface beam deflector. The angle-resolved optical scattering measurement was performed by mounting an InAsSb infrared detector (PDA10PT, Thorlabs Inc.) on a custom-made semi-circular slider track with an angular accuracy of 0.1°. The beam deflector device under test was placed at the center of the semi-circular track, and the zero-degree incidence angle and baseline laser power were calibrated by aligning the detector to the laser beam without the beam deflector in place. The laser beam was chopped at a frequency of 1,728 Hz. The chopped detector signal was transmitted to a lock-in amplifier (SR810, Stanford Research Systems, Inc.) and then recorded by a computer. The infrared image of the diffracted spots shown in Fig. 3e inset was taken by a liquid nitrogen cooled InSb focal plane array (FPA, Santa Barbara Infrared, Inc.).

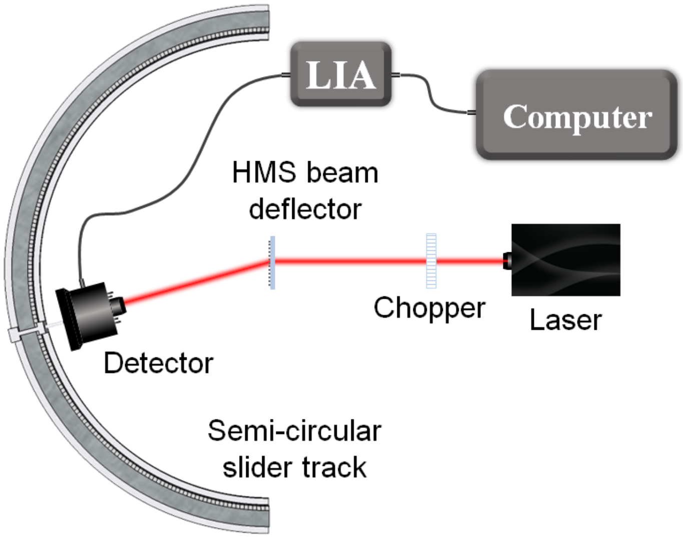

Supplementary Figure 6. Schematic diagram showing the measurement setup of the HMS beam deflector

Supplementary Note 5 – Cylindrical meta-lens measurement protocols and results

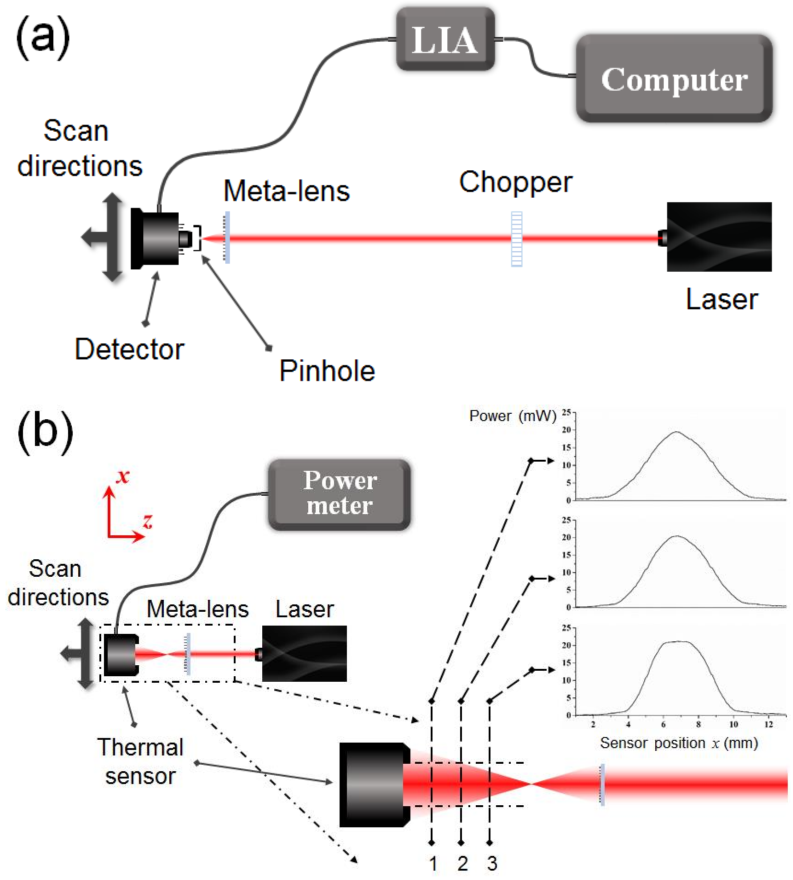

Supplementary Figure 7. Schematic diagrams of measurement setups for (a) mapping the output optical intensity profile from the HMS cylindrical lens; and (b) quantifying the numerical aperture of the cylindrical meta-lens.

Supplementary figure 7a sketches the setup for mapping the output optical intensity profile from the HMS cylindrical lens. The laser was normally incident on the cylindrical lens with its metasurface side facing the detector. A precision pinhole with a diameter of (5 ± 1) μm (P5S, Thorlabs Inc.) was attached to the front window of the detector (PDA10PT, Thorlabs Inc.) to limit the detector signal to only coming from light falling on the pinhole aperture. The detector was mounted onto a motion stage driven by two-axis servo actuators (TRA25CC, Newport Corporation). The intensity distributions shown in Figs. 4d-f were obtained by raster scanning the detector in the *x*-*z* plane. The scanning step size was fixed to 1 μm. Since the absolute distance between the pinhole and the lens surface can only be assessed with a measurement accuracy of a few tens of microns, all data collected using the pinhole scanning method (e.g. Figs. 4d-f and Fig. 4j) are referenced to the focal spot position at 5200 nm wavelength, which is taken as 500 μm away from the meta-lens, the design value. Nevertheless, the longitudinal chromatic aberration (slope of the line in Fig. 4j), which only hinges on the relative position of the focal spot, is accurate. It is also worth noting that the detector used in this experiment is thermo-electrically cooled to -30 °C and hermetically sealed in a package to prevent moisture condensation on the detector's active surface. As a result, the pinhole aperture is not in direct contact with the detector's active surface, leading to an effective reduction of the measurable numerical aperture to about 0.60.

To quantitatively assess the focusing quality, the focal spot profiles acquired using the pinhole scanning procedures were compared with simulation results. In the simulation, spatial distributions of light intensity at the focal plane of a diffraction-limited cylindrical lens were first calculated following the Huygens-Fresnel principle. The collected optical power through the pinhole was calculated by integrating the simulated light intensity at the focal plane over the pinhole aperture area. The results at three wavelengths (5110 nm, 5200 nm, and 5290 nm) are shown in Figs. 4g-i.

Since our HMS cylindrical lens has a designed NA of 0.71, we employed an alternative scheme illustrated in Supplementary figure 7b to accurately quantify its light collection ability. The laser was incident normally onto the device from the substrate side. Instead of using the single-element detector, we employed a large-area thermal sensor (S302, Thorlabs Inc.) to capture the output light from the meta-lens and the optical power collected by the sensor was monitored by a power meter (PM100D, Thorlabs Inc.). The thermal sensor was mounted on a two-axis linear motion stage. The insets in Supplementary figure 7b show three representative traces of the signal recorded as the thermal sensor position was scanned along the *x*-axis and at three different *z* positions with respect to the output beam (labeled as 1, 2, and 3, respectively). At position 1, the output beam width is larger than the width of thermal sensor's active area, and thus the sensor signal peaks when the sensor is aligned to the center of the beam. At position 3, the thermal sensor can intercept all the energy within the output beam from the meta-lens, and the *x*-scan signal trace exhibits a "flat-top" shape. The threshold position, labeled as "2" in the figure, coincides with the transition from a peaked trace to a "flat-top" one: this is when the beam width exactly equals the sensor's active area size. The lens numerical aperture was deduced from simple geometric relations of the distance between the thermal sensor and the lens as well as the width of the sensor's active surface.

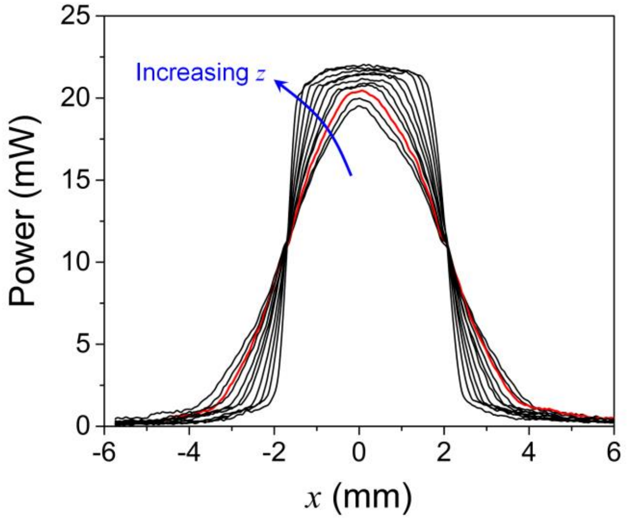

Supplementary Figure 8. Thermal sensor signal traces from the *x*-scans: the red curve corresponds to the threshold condition where the widths of the beam and the thermal sensor's active area equal.

Supplementary figure 8 displays a set of *x*-scan sensor signal traces from the NA measurement. The *z*-scan step size between the neighboring traces is 0.2 mm. The trace corresponding to the threshold position is marked with red color. The measurement yields a lens NA of (0.71 ± 0.01), in almost perfect agreement with the original design.

Supplementary Note 6 – Aspheric meta-lens measurement protocols and results

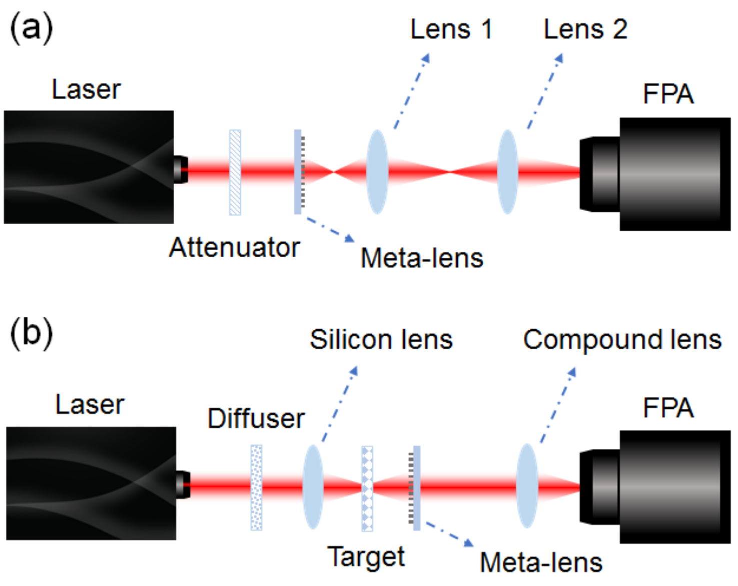

Supplementary Figure 9. Schematic diagram showing the measurement setup of the metasurface aspheric lens: (a) focal spot characterization; and (b) meta-lens imaging test.

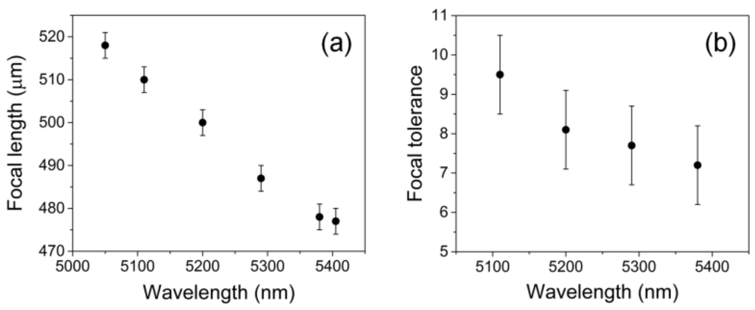

Supplementary Figure 10. (a) Focal lengths of the aspheric meta-lens measured at different wavelengths, where the error bar originated from the limited relative accuracy (± 2.5 μm) in determining the focal plane position; the absolute focal length values quoted have an uncertainty of 20 μm; and (b) focal tolerances, where the error bar originated from finite spatial resolution (± 1 μm) of the measurement setup.

Supplementary figure 9a illustrates the configuration for measuring the focal spot profiles of the aspheric meta-lens (Figs. 5d-i). The focal spot formed by the meta-lens was imaged onto a liquid nitrogen cooled InSb focal plane array (Santa Barbara Infrared, Inc.) via a microscope comprising Lens 1 (C037TME-E, Thorlabs Inc.) and Lens 2 (LA8281-E, Thorlabs Inc.). Lens 1 (an aspheric black diamond-2 lens) has a numerical aperture of 0.85, larger than that of the meta-lens to ensure that all light from the meta-lens focal spot was captured. The microscope provides an overall magnification of 80, which was calibrated using a USAF-1951 resolution target. The resolution target was fabricated in-house via lift-off patterning of thermally evaporated metal tin films on a double-side polished CaF_2 substrate. A similar setup was used to evaluate the focusing efficiency of the lens except that the focal plane array was replaced with a thermoelectrically cooled single-element detector (PDA10PT, Thorlabs Inc.) in conjunction with an optical iris (aperture diameter ~ 100 μm) and a lock-in amplifier to precisely quantify the total optical power within the aperture. We then take the encircled optical power falling within the second minimum of the Airy disk as the focused power. The fraction of the focused power over the total power within the aperture was quantified based FPA images taken over the entire aperture area. The focusing efficiency is defined as the ratio of the focused power to the total incident power on the meta-lens. We note that unlike the case of the cylindrical lens, divergence angle of the output beam from Lens 2 is reduced by the microscope such that the detector can collect all the optical power from the meta-lens focal spot.

The on-axis focal spot profiles (Fig. 5k) were recorded by translating the position of the meta-lens along the optical axis while fixing the locations of Lens 1, Len 2, and the FPA. The (absolute) Strehl ratio at each lens position along the *z*-axis was computed following protocols described in Supplementary Note 7. At each wavelength, the focal spot of the lens was taken as the on-axis location where the Strehl ratio reached maximum. Supplementary figure 10a plots the measured wavelength-dependent focal lengths of the aspheric meta-lens. The data suggest a longitudinal chromatic aberration of -0.12 μm/nm, close to that of the cylindrical flat lens (-0.11 μm/nm) as both types of lenses are constructed out of the same set of meta-atoms. The focal tolerance (focal depth) was defined as the *z*-range where the Strehl ratio is above 80% of the Strehl ratio at the focal plane. For instance, at 5200 nm the Strehl ratio gauged at the focal plane is very close to unity, and therefore the focal tolerance encompasses data points in Fig. 5l with Strehl ratios greater than 0.8. Supplementary figure 10b shows the measured focal tolerances at different wavelengths.

To test the imaging capability of the meta-lens, a setup illustrated in Supplementary Figure 9b was utilized. To suppress laser speckles, a single-side polished silicon wafers was inserted into the optical path as an optical diffuser to decrease spatial coherence of the illumination light. After passing through the diffuser, the laser light was focused by a silicon lens (LA8281-E, Thorlabs Inc.) to illuminate the USAF-1951 resolution target. The meta-lens was then deployed as the objective lens of an infrared microscope, and an off-the-shelf mid-IR compound lens (Asio Lens 40494-AA1, Janos Technology, LLC) acts as a tube lens to project the image onto the focal plane array.

Supplementary Note 7 – Quantifying focusing performance of the HMS aspheric lens

Focusing performance of the meta-lens is characterized by the Strehl ratio as well as the modulation transfer function (MTF). The Strehl ratio compares the generated focusing wavefront with a reference wavefront from a diffraction-limited lens. The (absolute) Strehl ratio S is defined as the ratio of the peak intensity of a measured PSF ($I_{measured}$) to the peak intensity of a PSF generated by an aberration-free lens with the same F/# ($I_{diffraction}$):

$$S = \frac{I_{measured}(x=0, y=0)}{I_{diffraction}(x=0, y=0)} \quad (7)$$

To calculate the Strehl ratio, the measured intensity profiles of the measured focal spot (Figs. 5d-i) are normalized such that the total optical power within a given area on the image plane equals that of the diffraction-limited focal spot[12]. An optical system with a Strehl ratio exceeding 0.8 is commonly regarded as diffraction limited (approximately $\lambda_0/4$ optical path difference)[13]. At each wavelength, the measured focal lengths (Supplementary Figure 10a) were used to calculate the corresponding diffraction-limited PSF. The Strehl ratios calculated from measurement data at the wavelengths of 5050 nm, 5110 nm, 5200 nm, 5290 nm, 5380 nm, and 5405 nm are shown in Fig. 5j.

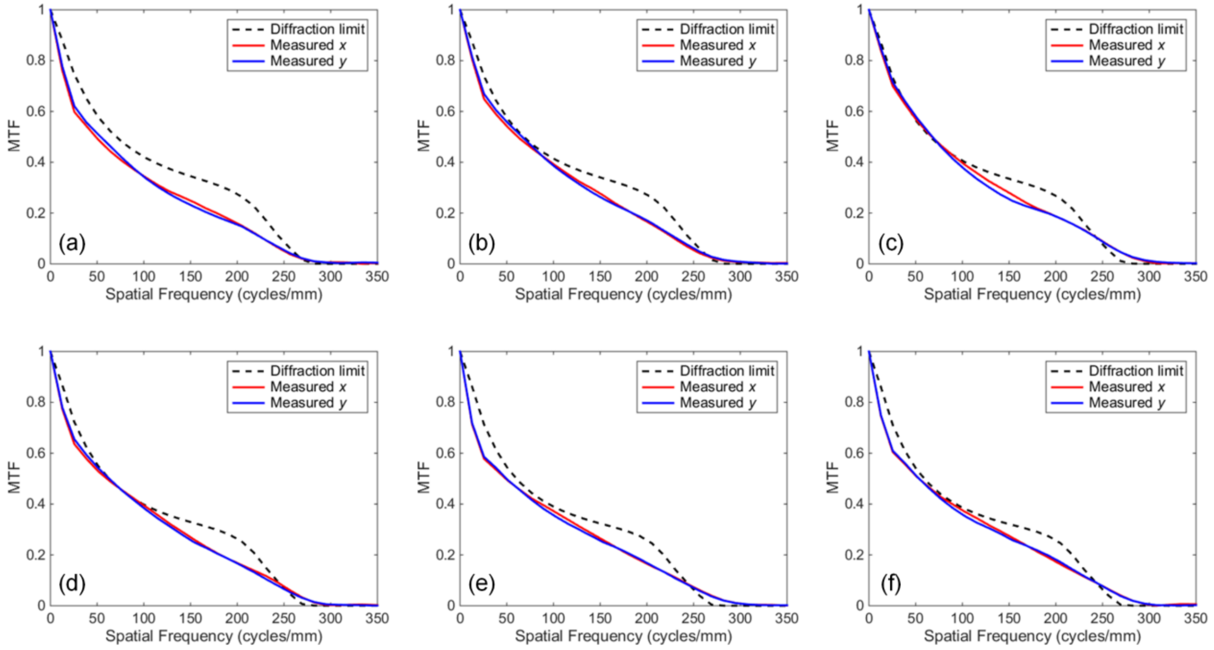

Supplementary Figure 11. ***x*- and *y*-sections of modulation transfer functions of the aspheric meta-lens at the wavelengths of: (a) 5050 nm; (b) 5110 nm; (c) 5200 nm; (d) 5290 nm; (e) 5380 nm; (f) 5405 nm. The diffraction-limited MTFs were computed based on an aberration-free ideal lens of the same aperture size as the meta-lens.**

Uncertainty in the Strehl ratio calculations primarily results from uncertainties in the focal length of the lens. We estimate that the absolute values of focal lengths presented in Supplementary Figure 10a have an error bar of ~ 20 μm, which translates to ~ 2% variation of the Strehl ratio based on our simulations.

MTFs of the meta-lens at multiple wavelengths were computed from Fourier transform of the measured focal spot profiles[14] (Figs. 5d-i) and displayed in Supplementary figure 11. The diffraction-limited MTFs plotted in the same figures for comparison were calculated for an aberration-free ideal lens of the same aperture size (1 mm by 1 mm) as the meta-lens. The diffraction-limited MTF simulations were performed using the Zemax software (Zemax, LLC) following the Huygens-Fresnel principle. We note that the Fraunhofer approximation is not valid in the model given the large NA of the meta-lens, and therefore the diffraction-limited optical transfer function of the lens cannot be straightforwardly computed by taking auto-correlation of the pupil function[15].

Supplementary Note 8 – Towards further enhancing optical efficiencies: loss mechanism analysis and new Huygens meta-atom designs

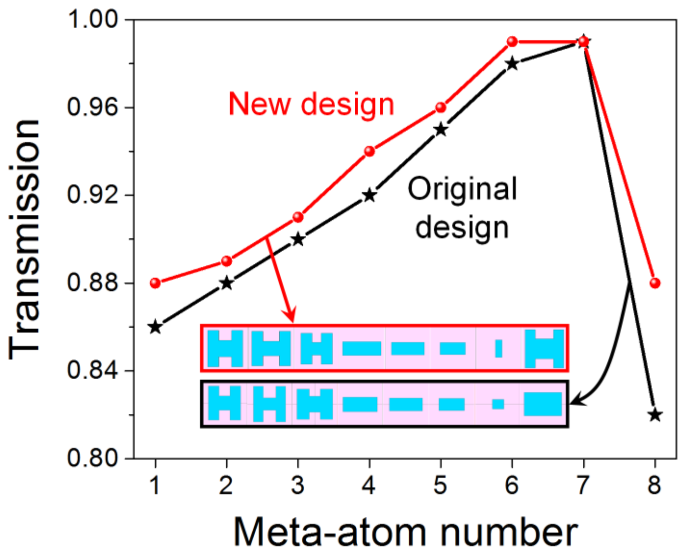

Supplementary Figure 12. Transmittance of: (red) the newly designed meta-atoms; and (black) the original design implemented in the fabricated devices; inset shows the top-view schematics of both designs.

In this section, we seek to estimate the sources of optical losses in the HMS devices and proposed means to further improve the efficiencies of these devices. First of all, all the devices we have measured are fabricated on CaF_2 substrates without anti-reflection (AR) coatings. By applying an AR coating to one-side of the substrate (the side without metasurfaces), the Fresnel reflection (up to ~ 3%) can be eliminated. We also compared the theoretical efficiencies of the beam deflector and the aspheric lens to those of experimentally measured values. For the beam deflector, the measured efficiency is ~ 7% lower than the theoretical calculation. For the aspheric lens, the discrepancy is ~ 5% (after taking into account the 3% Fresnel reflection loss). Therefore, the imperfect fabrication results in 5-7% efficiency loss.

We also examined the potential to further increase the theoretical efficiency limit based on the two-component meta-atom design. Supplementary figure 12 inset illustrates the new meta-atom design alongside the set currently used in the fabricated devices. In the new design, the same ultra-thin profile of the meta-atoms (650 nm thickness) is maintained and the meta-atom geometries are optimized to enhance their optical efficiencies. The main differences between the two designs include: 1) the original meta-atom #8, which has a considerably lower efficiency than other meta-atoms, is replaced with an H-shaped cell in the new layout; and 2) the dimensions of other atoms are slightly modified. Supplementary table 2 summarizes the new meta-atom structural dimensions, and Supplementary figure 12 compares the simulated optical transmission for the new design with that of the original meta-atoms. Our model shows that the new meta-atom design can boost the optical efficiencies of the HMS devices by an additional 4% to reach an overall efficiency of 87%.

Supplementary Table 2. Dimensions of new meta-atom designs with further improved efficiencies

Meta-atom number	1	2	3	4	5	6	7	8
L_x (L_{xd})	1.92 (0.7)	2.12 (0.9)	1.72 (0.5)	2.1	1.8	1.29	0.36	2.12 (1.0)
L_y (L_{yd})	2.0 (0.9)	1.9 (0.8)	1.7 (0.7)	0.75	0.69	0.72	0.96	2.1 (1.1)

Supplementary References

1 Harrington, R. F. Time-Harmonic Electromagnetic Fields. (2001).

2 Kuester, E. F., Mohamed, M. A., Piket-May, M. & Holloway, C. L. Averaged transition conditions for electromagnetic fields at a metafilm. *IEEE Transactions on Antennas and Propagation* **51**, 2641-2651 (2003).

3 Holloway, C. L., Mohamed, M. A., Kuester, E. F. & Dienstfrey, A. Reflection and transmission properties of a metafilm: With an application to a controllable surface composed of resonant particles. *IEEE Transactions on Electromagnetic Compatibility* **47**, 853-865 (2005).

4 Pfeiffer, C. & Grbic, A. Metamaterial Huygens' surfaces: tailoring wave fronts with reflectionless sheets. *Phys Rev Lett* **110**, 197401 (2013).

5 Kim, M., Wong, A. M. & Eleftheriades, G. V. Optical Huygens' metasurfaces with independent control of the magnitude and phase of the local reflection coefficients. *Physical Review X* **4**, 041042 (2014).

6 Kerker, M., Wang, D.-S. & Giles, C. Electromagnetic scattering by magnetic spheres. *J Opt Soc Am* **73**, 765-767 (1983).

7 Decker, M. *et al.* High-efficiency dielectric Huygens' surfaces. *Adv Opt Mater* **3**, 813-820 (2015).

8 Shalaev, M. I. *et al.* High-efficiency all-dielectric metasurfaces for ultracompact beam manipulation in transmission mode. *Nano Lett* **15**, 6261-6266 (2015).

9 Zhao, W. *et al.* Dielectric Huygens' Metasurface for High-Efficiency Hologram Operating in Transmission Mode. *Sci Rep-Uk* **6** (2016).

10 Pfeiffer, C. *et al.* Efficient light bending with isotropic metamaterial Huygens' surfaces. *Nano Lett* **14**, 2491-2497 (2014).

11 Yu, Y. F. *et al.* High-transmission dielectric metasurface with 2π phase control at visible wavelengths. *Laser Photonics Rev* **9**, 412-418 (2015).

12 Khorasaninejad, M. *et al.* Metalenses at visible wavelengths: Diffraction-limited focusing and subwavelength resolution imaging. *Science* **352**, 1190-1194 (2016).

13 Boreman, G. D. *Modulation transfer function in optical and electro-optical systems*. Vol. 21 (SPIE Press Bellingham, WA, 2001).

14 Aieta, F., Genevet, P., Kats, M. & Capasso, F. Aberrations of flat lenses and aplanatic metasurfaces. *Opt Express* **21**, 31530-31539 (2013).

15 Goodman, J. W. *Introduction to Fourier optics*. (Roberts and Company Publishers, 2005).
